# Invasion of Pheochromocytoma from the Caudal Vena Cava to the Right Ventricular Cavity in a Dog

**DOI:** 10.1155/2020/5382687

**Published:** 2020-02-11

**Authors:** Takafumi Machida, Noboru Machida

**Affiliations:** ^1^Department of Pharmacology, St. Marianna University School of Medicine, Kawasaki, Kanagawa 216-8511, Japan; ^2^Laboratory of Veterinary Clinical Oncology, Tokyo University of Agriculture and Technology, Fuchu, Tokyo 183-8509, Japan

## Abstract

Pheochromocytomas are catecholamine-secreting tumors that are composed of neuroectoderm-derived chromaffin cells. An 8-year-old miniature dachshund with abdominal distension was diagnosed with a neuroendocrine tumor with invasion from the caudal vena cava to the right ventricular cavity. The dog died due to hypotensive shock from the vagal reflex, and on autopsy, an extra-adrenal pheochromocytoma (paraganglioma) was diagnosed in the caudal abdomen. At autopsy, the tumor plug of the caudal vena cava was confirmed. To the best of our knowledge, this is the first case report that echo-captured the extension of pheochromocytoma in the right ventricle and shows it in a figure and video file.

## 1. Introduction

Pheochromocytomas are catecholamine-producing neuroendocrine tumors that arise from the chromaffin cells of the adrenal medulla or sympathetic paraganglia [1]. Dogs with pheochromocytomas show several vague and nonspecific clinical signs that are attributed to the excessive secretion of catecholamines [2]. Therefore, the diagnosis of pheochromocytoma is based on the biochemical detection of the tumor-based hormonal oversecretion. Typically, these tumors occur in older dogs, and common clinical signs, which result from catecholamine overproduction, include weakness, episodic collapse, lethargy, anorexia, tachypnea, panting, tachycardia, ascites, polyuria/polydipsia, hypertension-associated bleeding, vomiting, and diarrhea [1].

A considerable proportion of pheochromocytomas are discovered incidentally in clinically asymptomatic dogs [3, 4].

## 2. Case Presentation

An 8-year-old spayed female miniature dachshund, weighing 7.3 kg, was referred to our clinic with clinical signs of abdominal distension for five days prior to consultation. There was no history of trauma, and written consent was obtained from the dog's owner for the publication of this case report and accompanying images. On physical examination, the vital signs were normal (temperature: 38.4°C, pulse: 120 beats/min, respiratory rate: 36 breaths/min, and mucous membranes: pink and moist). No alopecia was noted. On abdominal palpation, there was ascites, and palpation of both elbows and the right hip showed tenderness and crepitation. Hematology and chemistry panels were normal, but the C-reactive protein level was elevated at 1.1 mg/L [5]. The pathophysiology of ascitic fluid was consistent with that of denatured transudate (specific gravity = 1.024; protein = 2.5 g/dL; cell number = 1000/*μ*l), and the cytology showed bare nuclei and rough chromatin.

**Figure 1:**
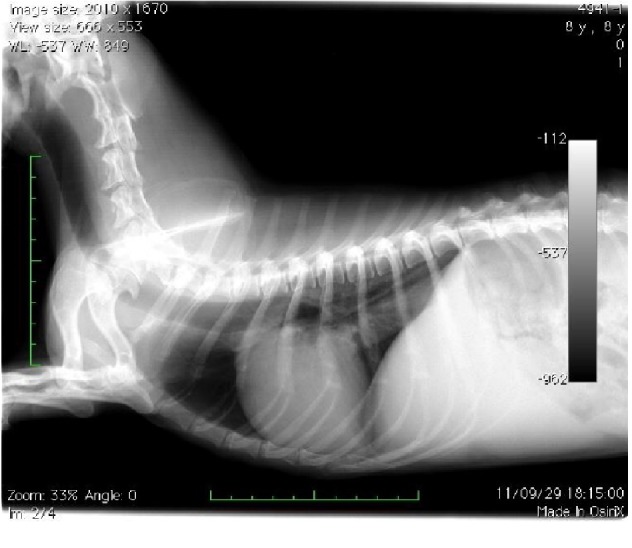
Lateral views showing enlargement of the caudal vena cava.

**Figure 2:**
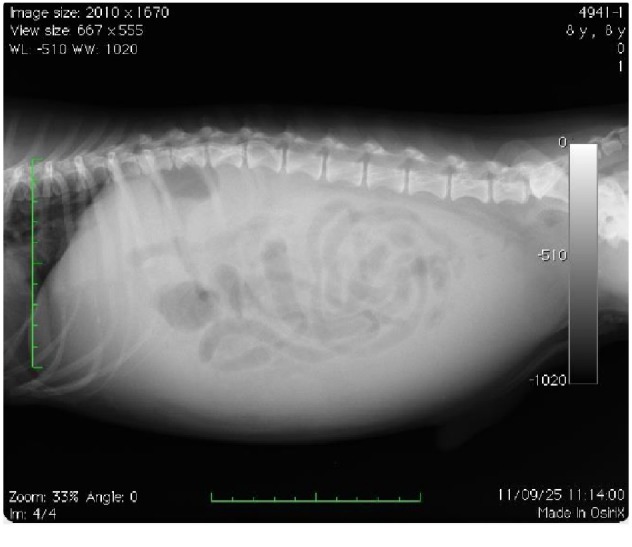
Abdominal radiograph showing ascites.

**Figure 3:**
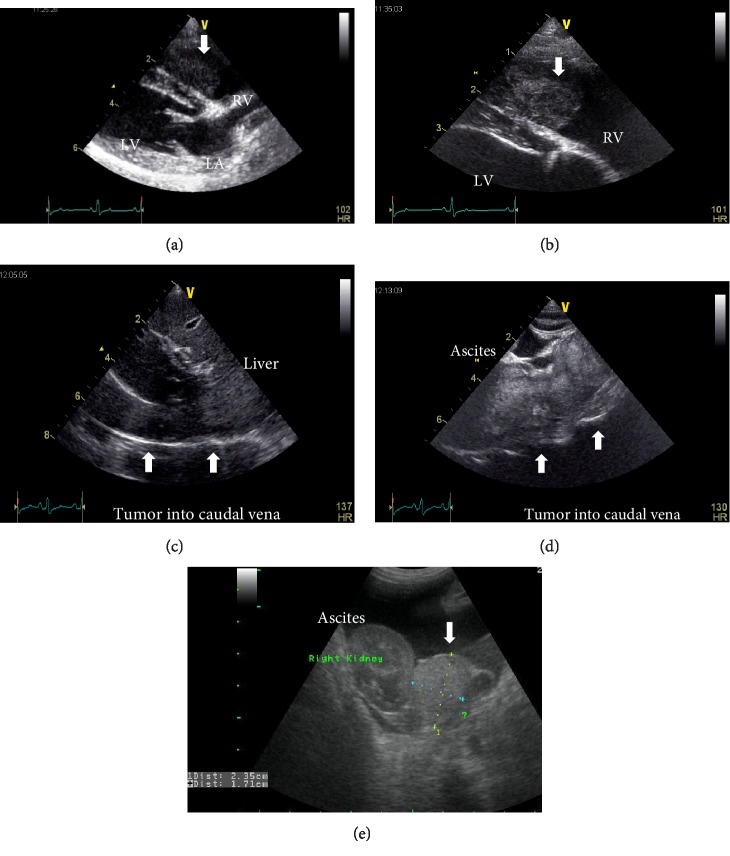
Transthoracic echocardiographs showing tumor invading into the right ventricular cavity on the right parasternal long axis four-chamber view (a). Tumor (arrow) can be seen within the right ventricle. Abdominal echocardiographs showing the tumor (arrow) invading into the caudal vena cava (b), (c), and the tumor and right kidney (d).

On thoracic auscultation, there was a Levine III/VI systolic murmur at the tricuspid valve orifice, but the electrocardiogram (*α*6000 AX-D, Fukuda M-E Kogyo Co., Ltd., Tokyo, Japan) was normal. The systolic blood pressure, which was measured using an oscillometric device (BP-100D, Fukuda M-E Kogyo Co., Ltd.), was 138 mmHg (normal systolic range: 100–160 mmHg; normal diastolic range: 60–100 mmHg), and the thoracic and abdominal radiographs showed a large caudal vena cava and ascites, respectively (Figures 1 and 2). Transthoracic echocardiography (Vivid E9, General Electric, Tokyo, Japan) showed mild tricuspid regurgitation (2.8 m/s), which was suggestive of an adrenal gland tumor between the atrium and ventricular cavity. The thoraco-abdominal echocardiography findings were also suggestive of a right adrenal tumor, which invaded into the right heart cavity, and the tip of the tumor had reached into the right ventricle (Figure 3(a)) and caudal vena cava (Figures 3(b) and 3(c)). The right ventricular systolic pressure calculated from the tricuspid regurgitation velocity was 36 mmHg (normal systolic range: 15–30 mmHg; normal diastolic range: 2–8 mmHg), and the other echocardiography findings were normal. The abdominal ultrasound showed the kidney and a 2.35 cm × 1.71 cm mass in the right cranial quadrant of the abdomen (Figures 3(d) and 3(e)), which was potentially localized within the root of the mesentery. No tumor was found in other organs on radiographs and echocardiography. There was no lymph node swelling. Fine-needle aspiration of the mass, which was performed under ultrasound guidance, showed suppurative and histiocytic inflammation with peripheral blood contamination. The pre-ACTH serum cortisol level was 5.7 *µ*g/dL (normal range: 2.0–6.0 *µ*g/dL), and the post-ACTH serum cortisol level was 11 *µ*g/dL (normal range: 6.0–18.0 *µ*g/dL). The dog was treated with supportive care, pheochromocytoma was suspected, and the owner was advised to give an *α* blocker, but drug treatment was refused.

At home, the dog seemed to have fallen while playing on foam. The dog probably died of hypotensive crisis 11 days after the hematology tests.

At necropsy, it was confirmed that the tumor plug of the posterior vena cava was due to an adrenal tumor that filled the vascular lumen rather than invading the vessel wall.

**Figure 4:**
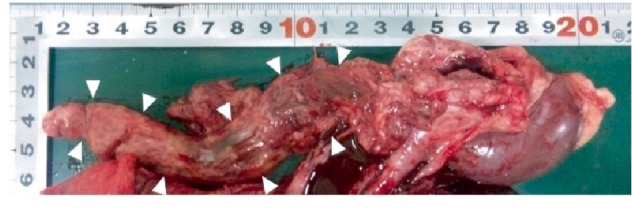
The right adrenal gland tumor (arrowhead) is ascending and progressive, with masses that have multiplied into the post cava, and the tip of the tumor reaches the right atrium.

The diagnosed tumor was ascending and progressive, with masses that multiplied into the post cava, and the tip of the tumor reached the right atrium (Figure 4) as the mass progressed into the right atrium.

Pheochromocytoma-related tumor block to the posterior vena cava is sometimes seen, but there has been only one previously published case [6].

**Figure 5:**
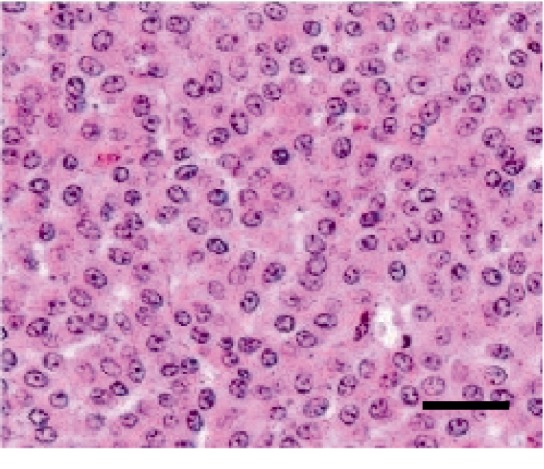
Histological sections of the neoplastic tissue of the right adrenal gland. Pleomorphic cells are divided into lobules by differing amounts of fibrovascular stroma and further divided by thin trabeculae of fibrous tissue into variably sized packets or nests. Hematoxylin eosin stain. Bar: 50 *µ*m.

At necropsy, an ovoid mass was located in the right adrenal gland. On sectioning, the mass was multilobular and were variegated light brown to yellowish red to pink due to areas of hemorrhage and necrosis. A small remnant of the adrenal gland was found at its periphery. The neoplastic tissue extended through the caudal vena cava into the right atrial lumen, forming a long and narrow thrombus. Histopathological examination revealed pleomorphic cells divided into lobules by relatively small amounts of fibrovascular stroma and further divided by thin trabeculae fibrous tissue into variably sized packets or nests (Figure 5). The predominant cell type was epithelioid in appearance and had cuboidal to polyhedral, lightly eosinophilic, finely granular cytoplasm of variable size and with poorly defined margins. Most of the neoplastic cells contained Grimelius silver-positive granules in the cytoplasm, indicative of a neuroendocrine cell origin. The nuclei varied in size and shape, but most were round to oval and usually located centrally with either fragmented or condensed (finely granular to largely clumped) chromatin and one to three prominent nucleoli. Although mitotic figures were infrequent, marked anisocytosis and anisokariosis were evident.

## 3. Discussion

To the best of our knowledge, this is the first case report that echo-captured the extension of a pheochromocytoma into the right ventricle and showed it in a figure and video file. Examination showed an enlarged vena cava and the presence of ascites. Invasion of the caudal vena cava in dogs with pheochromocytoma is common, and the resulting obstruction may cause ascites and hind limb swelling [1, 7]. At autopsy, it was seen that the tip of the tumor had reached into the right ventricle, which was later identified as tumor extension. Immunohistochemistry has no practical value for determining the tumour grade of canine. At autopsy, the tumor was easily and smoothly withdrawn from the posterior vena cava, and it was found that the pheochromocytoma did not invade the caudal vena cava wall. The same was noted in a previous case report [4], and when the tumor was pulled out by surgery, it is thought that the risk of death due to bleeding would be low. Thus, this case will likely be of interest to veterinary surgeons who work with companion animals.

## Data Availability

No data were used to support this case report.

## Supplementary Materials

Supplementary Materials Video files show transthoracic echocardiographs taken from the right parasternal long axis four-chamber view. Tumor invades into the right heart cavity, and the tip of the tumor has reached into the right ventricle.
